# Pitch chroma information is processed in addition to pitch height information with more than two pitch-range categories

**DOI:** 10.3758/s13414-022-02496-1

**Published:** 2022-06-01

**Authors:** Bernhard Wagner, Christopher B. Sturdy, Ronald G. Weisman, Marisa Hoeschele

**Affiliations:** 1 Acoustics Research Institute, Austrian Academy of Sciences, Vienna, Austria; 2 Department of Psychology, University of Alberta, Edmonton, Alberta Canada; 3 Neuroscience and Mental Health Institute, University of Alberta, Edmonton, Alberta Canada; 4 Department of Psychology, Queen’s University, Kingston, Ontario Canada

**Keywords:** Psychoacoustics, Hearing, Music cognition, Sound recognition

## Abstract

Octave equivalence describes the perception that notes separated by a doubling in frequency sound similar. While the octave is used cross-culturally as a basis of pitch perception, experimental demonstration of the phenomenon has proved to be difficult. In past work, members of our group developed a three-range generalization paradigm that reliably demonstrated octave equivalence. In this study we replicate and expand on this previous work trying to answer three questions that help us understand the origins and potential cross-cultural significance of octave equivalence: (1) whether training with three ranges is strictly necessary or whether an easier-to-learn two-range task would be sufficient, (2) whether the task could demonstrate octave equivalence beyond neighbouring octaves, and (3) whether language skills and musical education impact the use of octave equivalence in this task. We conducted a large-sample study using variations of the original paradigm to answer these questions. Results found here suggest that the three-range discrimination task is indeed vital to demonstrating octave equivalence. In a two-range task, pitch height appears to be dominant over octave equivalence. Octave equivalence has an effect only when pitch height alone is not sufficient. Results also suggest that effects of octave equivalence are strongest between neighbouring octaves, and that tonal language and musical training have a positive effect on learning of discriminations but not on perception of octave equivalence during testing. We discuss these results considering their relevance to future research and to ongoing debates about the basis of octave equivalence perception.

## Introduction

The octave is a musical interval defined as two notes separated by a doubling in frequency. The term “octave equivalence” (Burns, 1999; Patel, 2003) describes the phenomenon that notes separated by such a doubling in frequency sound similar – at times more so than notes with frequencies that are closer to one another (Allen, 1967; Hoeschele et al., 2012a, b; Kallman, 1982). The shared perceptual quality of frequencies separated by an octave is referred to as the “pitch chroma” of these notes. Pitch chroma, and thus the octave, is cross-culturally used as a common basis of pitch perception (Burns, 1999; Crickmore, 2003; but see Jacoby et al., 2019). The octave relationship is important not only to music but also to language learning: Young children as well as adults use the octave relationship in producing successful imitations of song or speech with a fundamental frequency outside of their own vocal range (Peter et al., 2008, 2009, 2015; see also Hoeschele, 2017, for a review). From the perspective of physics, this behavior makes sense because a note and its octave share as much overlap in harmonic frequencies as possible for two notes that do not share the same fundamental frequency (Hoeschele, 2017). This is because the octave of a given note contains every other harmonic of the given note. As such, when an individual cannot reproduce the fundamental frequency of a sound, octave transposing that sound results in the best possible imitation. Even in early ontogeny before the octave relationship is used in vocal imitation, its saliency appears to be present, as a study by Demany and Armand (1984) with infants at a pre-verbal developmental stage showed. Infants in this study exhibited stronger novelty responses to notes that were different from notes they were habituated to. This novelty response was smaller for notes an octave apart from a note they had previously been habituated to than for notes that differed from the habituated notes by other musical intervals. As such, it may come as no surprise that perception of octave equivalence has been suggested to be based in physiology (Braun & Chaloupka, 2006), with auditory midbrain neurons preferring harmonically related sounds (Langner & Ochse, 2006).

It may, however, be surprising that despite all the theory and observational data emphasizing the importance of octave equivalence, experimental demonstrations of octave equivalence in human adults have in the past proven to be difficult. Early studies found contradictory results using different procedures. Manipulation of single notes by octaves in simple melodies as originally used in Deutsch (1972) only showed an effect of octave when pitch contour was maintained (Dowling & Hollombe, 1977). Non-musical tasks showed octave equivalence only in trained musicians (Allen, 1967), not in non-musicians, a result also obtained by Krumhansl and Shepard (1979). Kallman (1982) found small or no effects of octave equivalence. These contradictory findings were summed up aptly by Burns (1999, p. 252) in the following statement: “If the results of some relevant experiments are accepted at face value, octave equivalence is shown by rats (Blackwell & Schlosberg, 1943), human infants (Demany & Armand, 1984), and musicians (Allen, 1967), but not by starlings (Cynx, 1993), 4- to 9-year-old children (Sergeant, 1983), or nonmusicians (Allen, 1967).”

Surveying the aforementioned studies, Hoeschele et al. (2012a, b) – just like Kallman (1982) did earlier – concluded that octave equivalence was only demonstrated where effects of pitch height were highly limited. Indeed, prior to Hoeschele et al. (2012a, b), only studies that used notes that were either exactly an octave or close to an octave apart succeeded in showing octave equivalence (e.g., Kallman, 1982). This emphasizes the importance of controlling for confounds of pitch height and octave equivalence when testing for the latter: As described above, two notes that are separated by an octave sound similar and potentially more so than notes with frequencies closer to one another (Allen, 1967; Hoeschele et al., 2012a, b; Kallman, 1982). However, similarity in pitch is also perceived when notes are close in pitch. On the whole, the closer two notes are in pitch the more similar they sound (see, e.g., Shepard, 1982). As such, the perception of octave equivalence and pitch height might oppose each other.

Hoeschele et al. (2012a, b) developed a standardized non-verbal operant procedure independent of music that successfully demonstrated octave equivalence in human adults. In this previous study, participants were trained to respond only to either the middle four or the outer eight notes (four lowest and four highest) of the 12 notes of octave four, but not to the remaining notes. When participants were presented with random unrewarded notes from octave five in a subsequent generalization test, they responded significantly more to the octave five notes that corresponded to the octave four notes they had previously been trained to respond to (Hoeschele et al., 2012a, b). To confirm that chroma was at the root of this response pattern in generalization, a transfer test was conducted after the test where the participants received additional training with both octaves. Participants were divided into two groups such that chroma was only a reliable cue for one of the groups. In both groups, the reinforcement pattern for the training octave remained the same as in the initial training octave. The reinforcement in the novel octave was where the two groups diverged: One group was presented with the same reinforcement pattern in the novel octave compared to the training octave, while the other group had exactly the reverse reinforcement pattern (i.e., if a C note was reinforced in the training octave it was not reinforced in the novel octave, and vice versa for the second group). Here participants who were in the reversed-pattern group had significantly flatter learning curves, at times even making errors in the initial training octave (Hoeschele et al., 2012a, b). As such, results in both tests of the procedure were exactly what would be expected if participants perceive notes separated by a doubling in frequency as similar. Hoeschele et al. (2012a, b) also provided validation for this experiment by replicating the outcome with slight variations in implementation (e.g., which notes were reinforced).

This non-verbal paradigm from Hoeschele et al. (2012a, b) is a highly useful tool as it is applicable with different human groups across barriers of language or speech impairment, and even across species (Hoeschele et al., 2013; Wagner et al., 2019). Yet, while the study provided a reliable and useful paradigm to test for octave equivalence, why it succeeded where others did not is not yet fully clear. A three-range task was used mainly due to conclusions drawn from Kallman (1982). Kallman (1982) could only document octave equivalence when the range of choices for “similar” notes was restricted in pitch height and far from the comparison note. Hoeschele et al. (2012a, b) hypothesized that this was the case because pitch height could not be used to meaningfully solve the task. Specifically, if pitch height is difficult to use, pitch chroma may become more salient. As such, Hoeschele et al., 2012a, b) decided to train participants on three ranges, because they supposed that when using two ranges the task may be solved by pitch height rather than chroma. The authors hypothesized that a task with more than two ranges would be difficult to solve with pitch height and as such force listening to chroma. Previous work by, for example, Njegovan et al. (1995) and Weisman et al. (2004, 2010) showed that three range tasks were possible to solve, whereas participants without absolute pitch could not solve tasks with a large number of ranges. As such, a three-range task was implemented as it was known that this was solvable for participants, while tasks with more than three ranges or even individual notes were rejected as being too difficult or impossible to solve. However, whether this difference between two-range and three-range tasks exists was not actually tested in Hoeschele et al. (2012a, b). Testing whether it does could lead to insights on when the use of pitch chroma is triggered in listeners and whether it is truly the case that pitch height is the more salient cue, applied more readily where possible.

As such, here we endeavoured to replicate and extend the results from the original study by testing further variations of the paradigm. Namely, we introduced two-range tasks in addition to the three-range task for generalization as well as transfer testing. The expectation here would be that with two ranges participants would respond by using pitch height as their only cue, responding either more or less the higher pitched a note is. Transfer training, just like in the original study, can further help us understand whether participants are using pitch height or chroma by either reinforcing or diminishing the usefulness of chroma and/or pitch height as a cue.

Furthermore, Hoeschele et al. (2012a, b), while verifying the procedure using several variations concerning amplitude and training patterns, only tested across two neighbouring octaves. But does octave equivalence occur beyond neighbouring octaves? In practice (e.g., in speech imitation – see Peter et al., 2008, 2009, 2015), generalizing across two octaves will not happen nearly as often as across one octave, and may only occur in contexts with musically trained individuals.

**Fig. 1:**
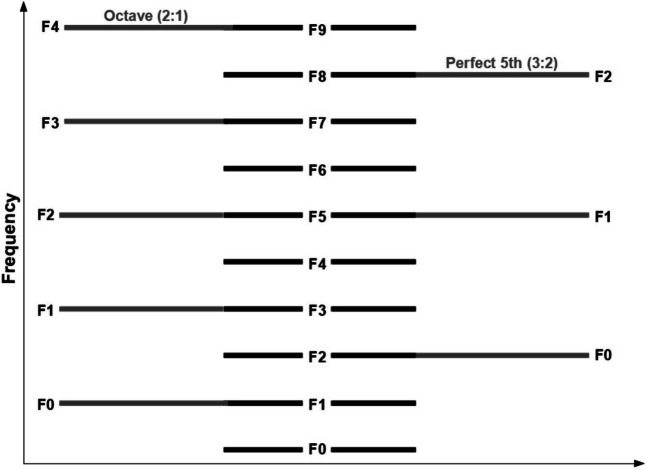
Overlap in harmonics of a sound (middle) and its octave (left) and octave plus perfect fifth (right)

In addition, if octave equivalence arises because of the similarity of harmonic content, then the interval of an octave plus a perfect fifth (two overtones above the fundamental) should sound more similar than a note two octaves away (three overtones above the fundamental). To explain this more precisely: Using the same F0 results in complete harmonic overlap. If this is not possible, using F0 transposed by an octave results in the second largest possible overlap, using F0 transposed by an octave plus a perfect fifth results in the third largest harmonic overlap, and using F0 transposed by two octaves results in the fourth largest harmonic overlap. As such, if overlap in harmonics of the human voice and other harmonic sounds are the key to octave equivalence perception, then octave equivalence may only occur (or occur more strongly) across neighbouring octaves. See Fig. 1 for a visualization of these relationships.

Finally, if octave equivalence perception is based on harmonics such as those found in the human voice, then it would make sense that it is universal. While octave equivalence is argued to be a musical universal (e.g., Brown & Jordania, 2013) this claim is based more on comparison of musical traditions rather than empirical evidence. Hove et al. (2009) did not test octave equivalence but found cross-cultural differences in pitch perception connected to culture but not to the ability to speak a tonal language. Could these differences extend to octave equivalence? A recent study by Jacoby et al. (2019) suggests that octave perception may not occur in the native Amazonian Tsimane'. As such, more testing of the possible cultural influence on octave equivalence is called for. Hoeschele et al. (2012a, b) did consider the role language may play in the perception of octave equivalence. However, there may not have been sufficient variety among participants for an effect of language abilities, speaking a tonal language, or cultural affiliation to emerge.

As such, together, understanding why a three-range task was effective, whether it is effective beyond neighbouring octaves, and what the cross-cultural implications of the task are, can help us to understand the universal underpinnings of the octave equivalence phenomenon. To answer these open questions, we collected data from a large pool of participants using variations of the original paradigm. More specifically we: (1) implemented comparison of two-range and three-range tasks to test the hypothesis that the simpler two-range tasks would result in participants paying attention to pitch height over octave equivalence; (2) tested across three octaves instead of two to test the hypothesis that octave equivalence could be demonstrated across multiple octaves to gain further insight into the potential role of harmonics for octave equivalence; and (3) collected detailed personal data on language and musical abilities from the numerous participants of varied cultural backgrounds to test whether there was an effect of musical training, ability to speak a tonal language, or of cultural affiliation.

## Method

*Note:* The last author was able to assemble data collected several (> 7) years ago from various research assistants and our deceased colleague (RGW) who had intended to write up the paper as first author. However, in many cases during the recent data curation, we were unable to locate data. Procedures also varied slightly across datasets, with slightly different numbers of trials for different participants. Below, we clarify where such differences exist. The data reported in this paper are for all participants where we could verify that they were a unique individual; where we could find all their training, testing, and survey data; and where we were certain which survey data (if there were conflicts – survey data was collected via pen and paper) belonged with which experimental data.

### Participants

In total, we have complete data from 140 participants who completed this study for course credit either at the University of Alberta (89) or at Queen’s University (51). Participants provided their ages and the details of their music and language training in written responses to a questionnaire. Each gave informed written consent, and the Research Ethics Boards at the University of Alberta and Queen’s University approved our protocols.

Based on a survey provided to all participants (see *Stimuli and procedure*), we gathered the following descriptive data about participants. Participants ranged in age from 17 to 24 years (M = 18.79), 50 were men, and 90 were women. Because musical (e.g., Allen, 1967; Krumhansl & Shepard, 1979) and language training (e.g., Deutsch et al., 2004; Pfordresher & Brown, 2009) are sometimes factors in music perception, we have provided more information about the participants’ histories. Forty-five participants had no formal music training. The other 95 musically trained participants had between less than 1 month of training to 22 years of training (M = 6.89 years). As having learned a tonal language can influence absolute and relative pitch perception (Deutsch et al., 2009; Hove et al., 2010), we also asked participants about their language abilities. Of all participants, 94 had English as their first language, 17 had a form of Chinese, nine had Korean, and the remaining 20 participants had 14 other first languages. Overall, 69 participants (including all non-native English speakers) were at least bilingual. Six of the participants were trilingual.

### Apparatus

Training and testing were conducted on either a Toshiba 149 Tecra laptop (Intel Pentium M processor and Intel 855 series chipset) or a custom-built computer (components: Intel Core i7 930 CPU, Asus P6T SE motherboard, Creative Soundblaster Audigy SE sound card with a 100dB signal-to-noise ratio and frequency response < 10–40 kHz) using either Sennheiser HD 280 or Sennheiser HD 580 headphones (Sennheiser Canada, Montreal). The participants used a mouse to make their responses and could use a rotary control on the computer to adjust the volume to the headphones at any time during the experiment. The procedures and data collection were programmed in Visual Basic.

### Stimuli and procedure

All participants first read and signed an informed consent form and then completed a survey about their music and language background. Afterwards, they completed four testing phases: absolute pitch testing, operant discrimination training, generalization testing, and transfer training. During all four testing phases, any questions about the practical aspects of completing the experiment were answered, but participants were told that theoretical questions about the experiment would be answered at the end, and to just do their best. After completing all four testing phases, participants were given a debriefing form with information about the goals of the experiment and any remaining questions were answered.

### Absolute pitch testing

The protocol was adapted from a procedure used by Athos et al. (2007) to test 2,213 participants: The note durations and frequencies were a direct replication of Athos et al.’s procedure. In the note-naming tests, we identified AP possessors using Athos et al.’s scoring protocol: 1 point for each correct identification, and 0.75 points for responses to notes ±1 semitone from the correct note.

Sine-wave tones presented in the test were all 440 ms long and synthesized at the frequencies of 40 notes randomly sampled from the 66 notes on the chromatic scale that spans the 5 1/2 octaves from C2 to A#8, on the basis of A4 = 440 Hz; each note was played for 1,000 ms (see Athos et al., 2007). The actual notes presented were D#2 (77.8 Hz), F2 (87.3 Hz), F#2 (92.5 Hz), G#2 (103.8 Hz), A#2 (116.5 Hz), B2 (123.5 Hz), C#3 (138.6 Hz), D#3 (155.6 Hz), E3 (164.8 Hz), F3 (174.6 Hz), G3 (196 Hz), G#3 (207.7 Hz), C4 (261.6 Hz), C#4 (277.2 Hz), D4 (293.7 Hz), D#4 (311.1 Hz), F4 (349.2 Hz), F#4 (370 Hz), A4 (440 Hz), C5 (523.3 Hz), C#5 (554.4 Hz), D5 (587.3 Hz), E5 (659.3 Hz), F#5 (740 Hz), G5 (784 Hz), G#5 (830.6 Hz), A5 (880 Hz), A#5 (932.3 Hz), C6 (1046.5 Hz), D6 (1174.7 Hz), A6 (1760 Hz), B6 (1975.5 Hz), C#7 (2217.5 Hz), D#7 (2489 Hz), F#7 (2960 Hz), B7 (3951.1 Hz), E8 (5274 Hz), F#8 (5919.9 Hz), G8 (6271.9 Hz), A#8 (7458.6 Hz).

These tones and all the others presented in this study were constructed at a standard 16-bit, 44.1-kHz sampling rate and ramped at onset and offset, respectively, upwards and downwards for 5 ms. Because four of the sine-wave tones lie above the notes on the piano keyboard (in octave 8) and proved difficult to identify, participants rarely named them accurately. In practice, therefore, the test consisted of 36 notes (see Athos et al., 2007).

The test began after a short practice session (eight trials), given to acquaint participants with making mouse responses to graphics on the screen and to allow participants to individually adjust the tone amplitude to a comfortable level. During the practice session and the test, a participant clicked on the “Play” button at the top of the screen and heard a tone selected randomly without replacement from the 40 test tones, which controlled for any possible predictable relative pitch carryover effects between tones (Ward & Burns, 1982). To “name” the musical note corresponding to a tone, the participant clicked on one of 12 black and white piano keys shown on the screen. The test continued without feedback until the participant heard all 40 tones. In this note-naming test and all following tests, the participants could take as much time as they liked between trials, as a trial began only after the participant had clicked the “Play” button.

### Operant discrimination training

Similar to the AP test, during the operant discrimination training phase participants could press a “Play tone” button to initiate each trial. However, now one of the 12 notes from the fourth octave of the 12-tone chromatic scale was presented randomly without replacement on each trial, until all 12 notes had been heard. Then all 12 notes were added back to the stimulus pool. Six of these notes were treated as rewarded or “S+” notes and six as unrewarded or “S-” notes. Participants were asked to classify notes into two categories (go and no-go tones) to the best of their ability, without any instructions about which notes made up each category. Participants were told that discrimination training was a test of their perceptual categorization ability but not that it was a test of octave equivalence.

Participants initiated a trial by clicking the button labeled “Play tone” on the screen to hear a tone. If a participant clicked on the button on the screen labeled “S+” after hearing a go tone, the word “correct” appeared in a box adjacent to the S+ button. If the participant clicked the S+ button on a no-go trial, the word “incorrect” appeared in a box adjacent to that button, and the next trial was delayed by 3 s. If a participant failed to click the S+ button after either a go or a no-go tone, the trial terminated after 2 s without feedback, as is typical in go/no-go discrimination procedures.

Each note was presented either 30 or 36 times in a random order without replacement for a total of 432 trials. Half the presentations of each note were at 70 dB and half at 80 dB to diminish the usefulness of amplitude as an auxiliary cue to make pitch-based ratings (Moore, 2013, p. 134-137). Participants were always presented with all 12 notes of the 12-tone chromatic scale from the fourth octave, but which tones were rewarded was not the same for all participants. Participants were divided into three groups: (1) -3+6-3, (2) +6-6, or (3) -6+6. Division into groups was done quasi-randomly to ensure a similar number of participants in all groups. The names reflect the categories of the 12 training notes in ascending order. For example, the -3+6-3 group were rewarded for responding to the middle six notes of octave four (D#, E, F, F#, G, G#) but not the outer three notes on either end (C, C#, D, and A, A#, B). For this group only, the octave was divided into three ranges. We did not have a counterbalanced version here (middle range unrewarded, outer ranges rewarded), because counterbalanced versions had already been used in Hoeschele et al. (2012a, b) and the results were the same. In the other two groups, the octave was divided in half, and either the lower notes were rewarded (+6-6) or the higher notes were rewarded (-6+6).

The actual stimuli presented were: C4 (261.6 Hz), C#4 (277.2 Hz), D4 (293.7 Hz), D#4 (311.1 Hz), E4 (329.6 Hz), F4 (349.2 Hz), F#4 (370 Hz), G4 (392 Hz), G#4 (415.3 Hz), A4 (440 Hz), A#4 (466.2 Hz), B4 (493.9 Hz).

### Generalization testing

The generalization phase was the same as the discrimination phase except that now all 36 notes from octaves four, five, and six were included. There were either 10 or 12 trials for each note. As such, the notes presented were: C4 (261.6 Hz), C#4 (277.2 Hz), D4 (293.7 Hz), D#4 (311.1 Hz), E4 (329.6 Hz), F4 (349.2 Hz), F#4 (370 Hz), G4 (392 Hz), G#4 (415.3 Hz), A4 (440 Hz), A#4 (466.2 Hz), B4 (493.9 Hz), C5 (523.2 Hz), C#5 (554.4 Hz), D5 (587.3 Hz), D#5 (622.2 Hz) E5 (659.3 Hz), F5 (698.5 Hz) F#5 (740 Hz), G5 (784 Hz), G#5 (830.6 Hz), A5 (880 Hz), A#5 (932.3 Hz), B5 (987.6 Hz), C6 (1046.5 Hz), C#6 (1109 Hz), D6 (1175 Hz), D#6 (1245 Hz), E6 (1319 Hz), F6 (1397 Hz), F#6 (1480 Hz), G6 (1568 Hz), G#6 (1661 Hz), A6 (1760 Hz), A#6 (1865 Hz), B6 (1976 Hz).

**Fig. 2:**
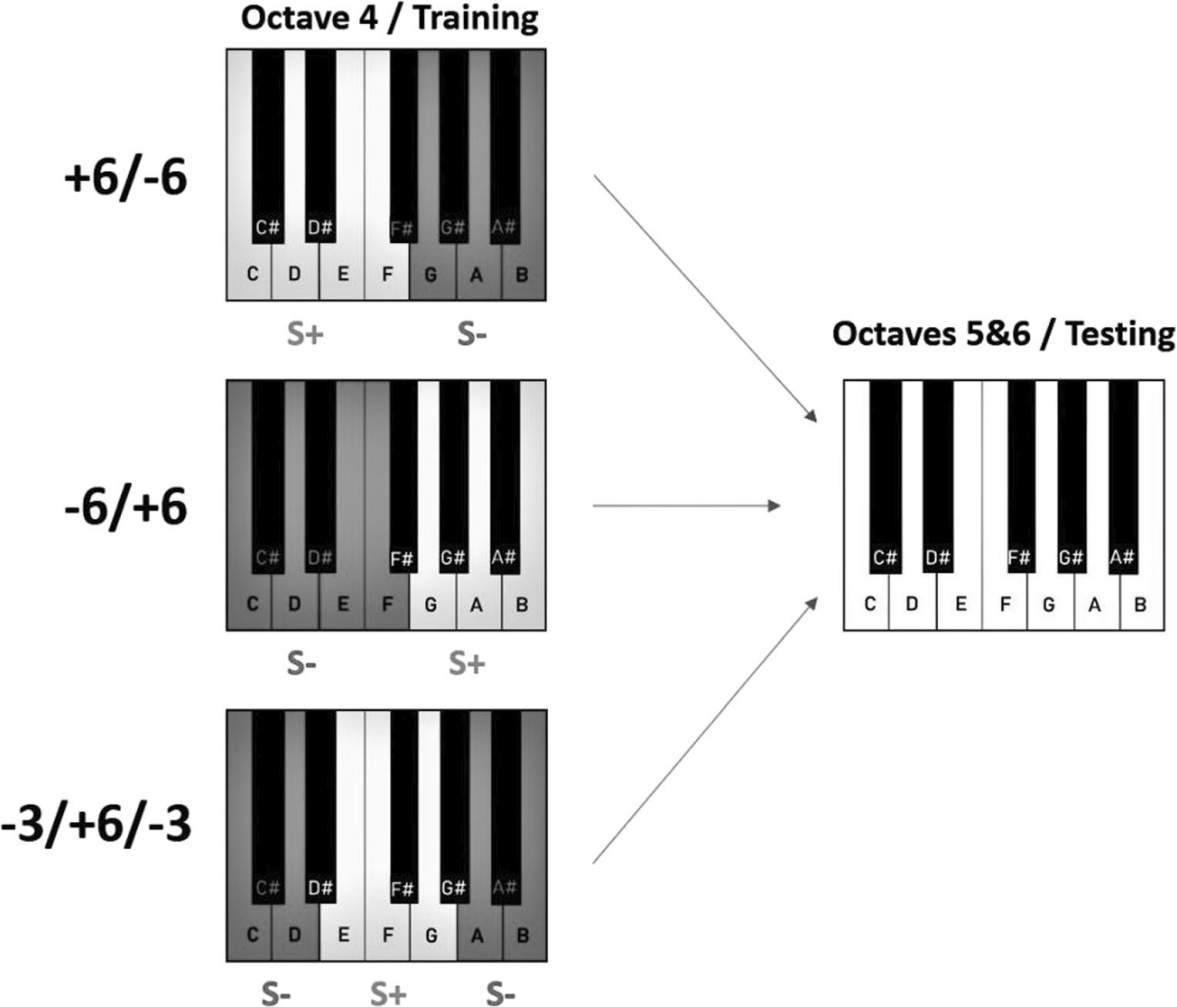
Patterns of reward in training octave four with subsequent test of unrewarded octave five and six testing notes. Dark grey areas on the keyboard and “S-“ denote non-rewarded notes, light grey areas and “S+” signify rewarded notes

Each note was played 10 or 12 times. No feedback was provided for responses during this phase. The participants were told that they would no longer receive feedback for their responses but were asked to respond as they had during training, to the best of their ability. Participants received no further instructions. See Fig. 2 for a visual representation of the training and testing process.

### Transfer training

**Fig. 3:**
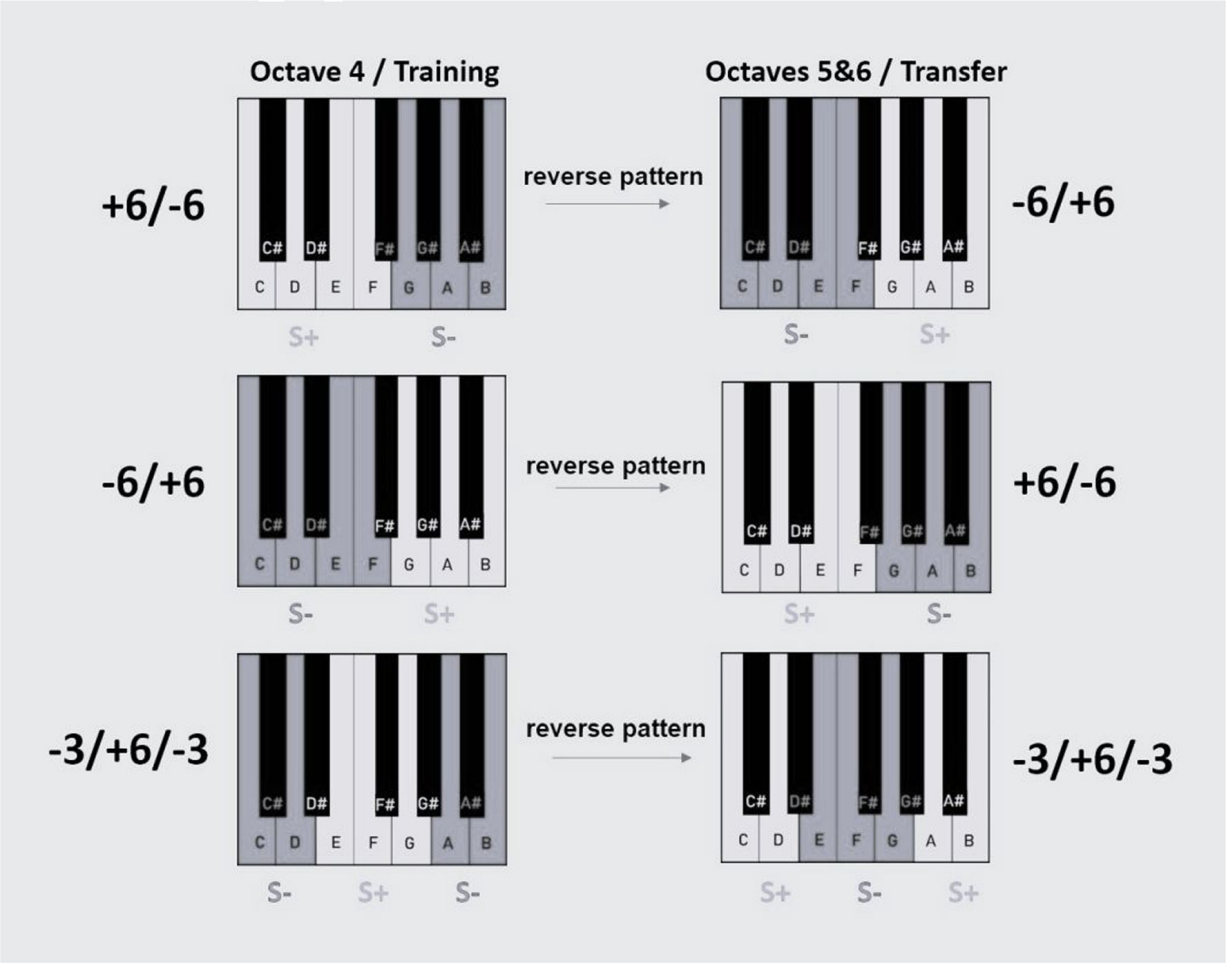
Reward contingencies for octave four and in octaves five and six for the reversed groups during transfer test. The matched groups had the same reward contingencies in octaves five and six as in octave four. Dark grey areas on the keyboard and “S-” denote non-rewarded notes, light grey areas and “S+” signify rewarded notes

This phase was the same as the discrimination phase except that it included all the notes from generalization testing (that is octaves four, five, and six) instead of only notes from octave four. There were either 10 or 12 trials per note. Participants were now provided feedback for their responses. For all participants, feedback to the notes in the fourth octave remained the same as during training. Participants from each group were further divided into two subgroups: matched and reversed. For the matched group, notes in octaves five and six had the same reward contingencies as those in octave four. For example, in the -3+6-3 group, notes D#, E, F, F#, G, and G# were rewarded in all octaves, but notes C, C#, D, and A, A#, and B were not. For the reversed group, reward contingencies in octaves five and six were reversed relative to octave four. For example, in the -3+6-3 group, notes D#, E, F, F#, G, and G# were rewarded in octave four but not rewarded in octaves five and six, while notes C, C#, D, and A, A#, and B were not rewarded in octave four, but rewarded in octaves five and six. See Fig. 3 for a visual representation of groups and their matched and reversed patterns for octaves above octave four.

### Statistical analysis

All analyses were conducted in R (Version 4.0.3, © GNU GPL) using RStudio (Version 1.3.1093, © GNU GPL) with no additional packages installed.

To analyse our data, we used logistic regressions as logistic regression is the standard analysis for modeling probability of binary target variables in terms of covariates (Fahrmeir et al., 2013). To control for potential confounding variables, we performed multivariable logistic regressions (R function “glm”, family = binomial).

To analyse the generalization test data, we used two types of logistic regressions:

- A multivariable logistic regression using the target variable “proportion of correct responses” with covariates sex, age, ability to speak a tonal language, and years of musical training for each octave separately. “Proportion of correct responses” here means the proportion of trials where participants responded by clicking the “S+” button for S+ stimuli, but for S- stimuli it is the proportion of trials where participants did not click on the “S+” button.
- A multivariable logistic regression using the target variable “proportion of responses” with covariates sex, age, ability to speak a tonal language, years of musical training, octave, range, and an interaction of octave and range. “Proportion of responses” here means the proportion of trials where participants clicked on the “S+” button on the screen for each stimulus regardless of whether a stimulus was S+ or S-.

We ran these two separate logistic regressions for each group as they allowed us to investigate two different aspects of the participants’ reaction in the generalization test in a way that would be impossible if we used only one of the two exclusively. The comparisons for analysis (a) with target variable “proportion of correct responses” give us a measure of overall accuracy of discrimination. By analysing effects of the covariates this further allows us to infer whether certain subgroups (i.e. tonal language speaking participants, musically trained participants, older/younger participants, and participants of one sex) outperformed other participants. However, analysis (b) with target variable “proportion of responses” allows us to determine whether a chroma or a pitch height strategy was used to solve the task. This is because analysis (b) allows for paired comparisons across ranges. As the core idea of this paradigm is that notes separated by an octave should be treated as similar by octave equivalence perceiving participants, we need to show that there is a significant difference between responses between S+ and S- ranges. Using *correct* responses as target variable is not useful here, for example, if a participant discriminates with 100% accuracy there would be no difference between S+ and S- range in correct responses whereas the actual responses are ten clicks for S+ and 0 clicks for S-. As such, here, only analysis of proportion of responses can successfully reveal octave equivalence.

In a further analysis of generalization test data we used Wilcoxon signed-rank tests (after using Shapiro-Wilk tests that found the data were not normally distributed) for comparison between proportion of responses to individual notes. P-values for these comparisons were Bonferroni corrected.

To analyse transfer test data we once more used a multivariable logistic regression with target variable: “proportion of correct responses” and covariates sex, age, ability to speak a tonal language, years of musical training, and a covariate for transfer type (i.e., whether a participant was in the “matched” or “reversed” condition). This analysis was conducted separately for each octave. Using this analysis exclusively was sufficient here because we were interested just in overall accuracy because practice time was insufficient for most participants to complete learning the new discrimination.

We treated results concerning data obtained from the survey (as opposed to the measured responses in the experiment) as exploratory and did therefore not correct p-values for multiple testing in analyses of this data.

## Results

In total, the 140 participants that we were able to include (see note at the beginning of the *Methods* section) were run in the following groups (parentheses denote the number of participants remaining after removing non-learners and absolute pitch possessors– defined below) see Table 1:

**Table 1:** The number of participants in each group. The numbers in brackets are the participants remaining after removing non-learners and absolute pitch possessors

	-3+6-3 Group	+6-6 Group	-6+6 Group
Matched	28 (27)	20 (20)	21 (21)
Reversed	23 (22)	32 (31)	16 (14)
Total	51 (49)	52 (51)	37 (35)

### Absolute pitch (AP) testing

Using Athos et al.’s (2007) AP criterion score (≥ 24.5), we found two AP possessors (scores 26 and 29) and 138 non-possessors (7.174 ± 2.51, max score 13.5). One AP possessor was in the -3+6-3 matched group, and one was in the -6+6 reversed group. We removed these AP possessors from analysis.

### Discrimination training

Most (n = 135) but not all participants (n = 3) learned the task. We defined learners based on responding more on average to the S+ range than the S- range (for the two-range groups) or either S- range (for the three-range group). Because there were so few non-learners, we removed these non-learners from further analyses. There was only one non-learner in each group, suggesting that our criterion for learners was equally achievable in all groups. We conducted a logistic regression in R-Studio as detailed above in the *Statistical analysis* section. We set -3+6-3 as the baseline group and found that there were significantly fewer correct discriminations in the -3+6-3 group compared to the +6-6 group (logistic regression: p < 0.001) and the -6+6 group (logistic regression: p < 0.001). We also found significant effects for years of musical training (logistic regression: p < 0.001) and for sex (logistic regression: p < 0.001) and age (logistic regression: p = 0.02651) with more trained, older, and female participants discriminating more accurately. However, there was no significant effect for the ability to speak a tonal language (logistic regression: p = 0.354).

### Generalization testing

To analyze the results from generalization testing we performed a multivariable logistic regression with target variable “percent of correct responses to a range within an octave” to test overall accuracy among participants and groups as detailed above in the *Statistical analysis* section. There were main effects for group and – just as for discrimination training – we set -3+6-3 as the baseline group and found that there was a significant difference in percentage of correct discrimination compared to the two-range groups in octaves four (logistic regression: all ps <0.001) and five (logistic regression: all ps < 0.001) and to the 6-6+ group in octave six (logistic regression: p = 0.0353) with the -3+6-3 group outperforming the other groups in octave four and the -6+6 group performing better than the other two groups in octaves five and six. There was no significant effect for speaking a tonal language (logistic regression: p = 0.0727 for octave four responses, p = 0.743 for octave five responses, p = 0.83446 for octave six responses).

**Fig. 4:**
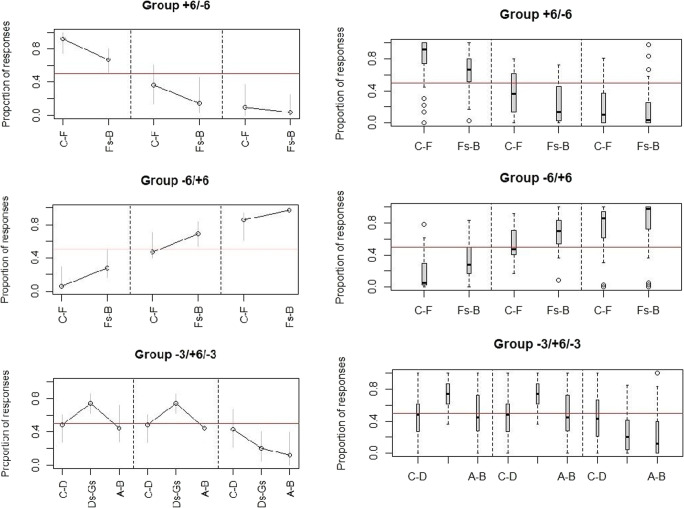
**Left:** Proportion of responses to note ranges for the three respective groups for all three octaves (four to six from left to right) in the generalization test. **Right:** Proportion of responses visualized as boxplot to give an estimate of data spread. For both, the grey bars lines denote the interquartile range and the red line denotes chance level

Additionally, we conducted a multivariable logistic regression comparing responses to the different ranges with the target variable “total number of responses” for each group separately to test whether participants responded according to a pitch chroma or a pitch height rule as detailed above in the *Statistical analysis* section. For each group the interaction of range and octave was significant at p < 0.001. We found effects as follows: In group +6-6 there were significant differences between the S- and the S+ range in octave four (logistic regression: p < 0.001) with more responses to the S+ range, between the octave four S- range and the corresponding ranges in octaves five (logistic regression: p = 0.0049) and six (logistic regression: p < 0.001) with fewer responses to the S- ranges in the higher octaves. There was also a significant difference between the octave four S+ range and the corresponding ranges in octaves five and six (logistic regression: all ps < 0.001) with fewer responses to the higher octave S+ ranges. These differences can also be seen in Fig. 4. We also found significant effects of amount of musical training (logistic regression: p < 0.001), age (logistic regression: p < 0.001), sex (logistic regression: p = 0.0041), and years of musical training (logistic regression: p < 0.001) on total number of responses with more musically trained, younger, female, and non-tonal language speaking participants pressing the button more often.

In group -6+6 there was a significant difference between the octave four S- and the S+ range (logistic regression: p < 0.001) with fewer responses to the S- range. There were significant differences between the octave four S- range and the corresponding ranges in octaves five and six (logistic regression: all ps < 0.001) with more responses to the S- ranges in the higher octaves. There were also significant differences between the octave four S+ range and the corresponding ranges in octaves five and six (logistic regression: all ps < 0.001) with more responses to the S+ ranges in the higher octaves. These differences can also be seen in Fig. 4. We also found a significant effect of sex and years of musical training (logistic regression: p < 0.001) with more musically trained and female participants pressing the button more often. Here we found no effect for age or speaking a tonal language (logistic regression: both ps > 0.05).

In group -3+6-3 there was a significant difference between the octave four S+ range and both S- ranges (logistic regression: both ps < 0.001) with more responses to the S+ range. There were also significant differences between the octave five S+ and S- ranges (logistic regression: p = 0.02 for the lower S- range; p < 0.001 for the higher S- range) with more responses to the S+ range. There were no significant differences between the two octave four S- ranges (logistic regression: p = 0.89625). There were also no significant differences between the octave four lower S- range and the octave five (p = 0.36106) and octave six lower S- range (logistic regression: p = 0.31712). There was a significant difference between the octave four higher S- range and the octave six higher S- range (logistic regression: p < 0.001), with more responses to the octave four higher S- range. There was no significant difference between the octave four higher S- range and the octave five higher S- range (logistic regression: p = 0.241). These differences can also be seen in Fig. 4. There were also significant effects of age, years of musical training, and ability to speak a tonal language (logistic regression: all ps < 0.001) with more musically trained, younger, and non-tonal language speaking participants pressing the button more often. There was no significant effect for sex (logistic regression: p > 0.05).

### Transfer test

**Fig. 5:**
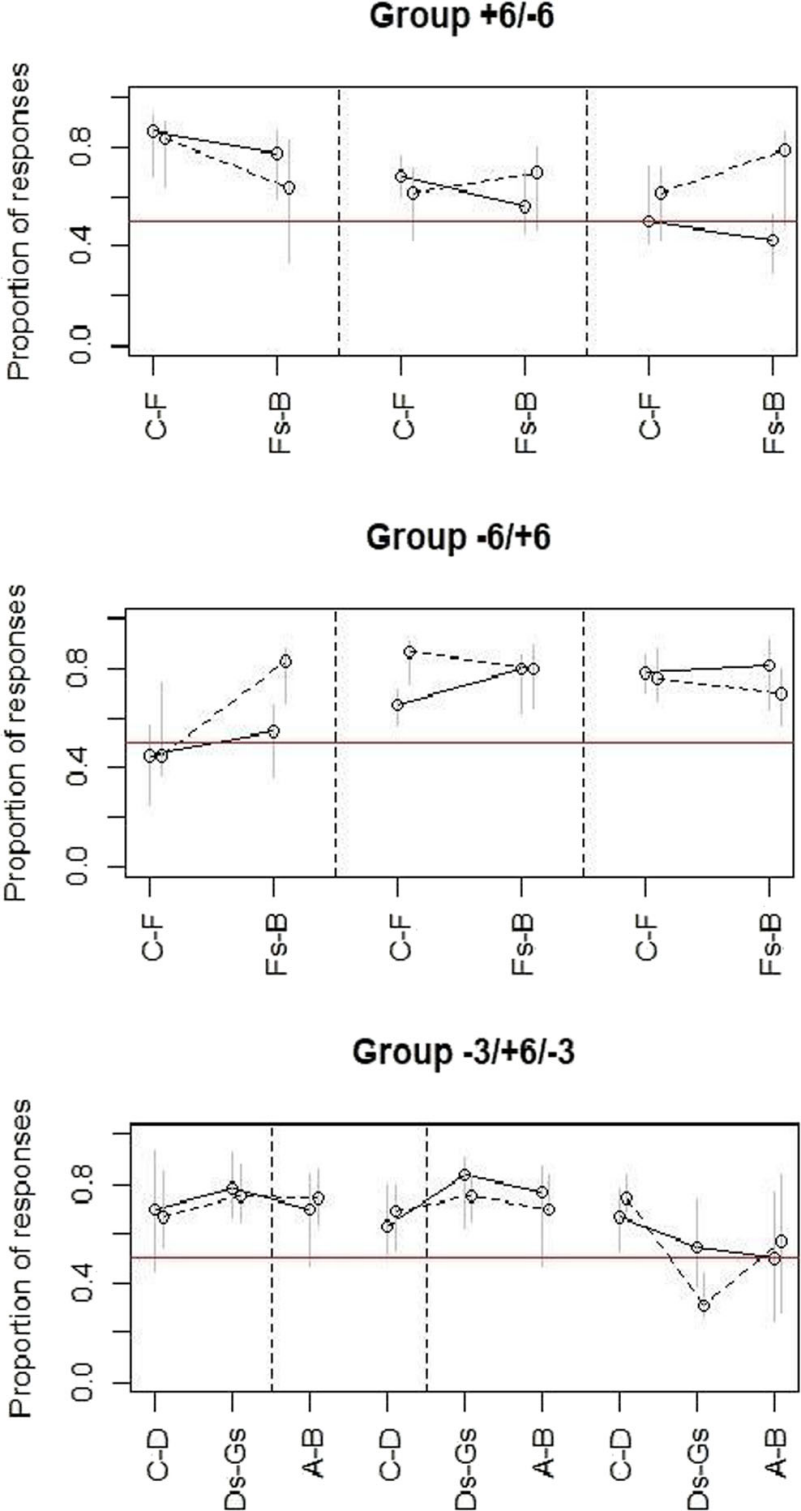
Median proportion of responses to note ranges for the three respective groups for all three octaves (four to six from left to right) in the transfer test. The grey bars denote the interquartile range. The dotted line is the reversed group. The red line denotes chance level

To analyze the results from the transfer test we once more performed a multivariable logistic regression with target variable “percent of correct responses to a range within an octave” to test overall accuracy among participants and groups as detailed above in the *Statistical analysis* section. Just as for discrimination training, we set -3+6-3 as the baseline group and found that there was a significant difference in percent of correct discrimination between group -3+6-3 and the other two groups in octaves four and five (logistic regression: both ps < 0.001) and in octave six (logistic regression: p = 0.02), with participants in the -3+6-3 group achieving a lower percentage of correct discrimination. In octave four there was a significant effect for sex (logistic regression: p = 0.0207) and ability to speak a tonal language (logistic regression: p < 0.001), with female participants and speakers of a tonal language achieving more accurate discrimination. There were no significant effects for age, years of musical training, or transfer type (logistic regression, all ps > 0.05). In octave five there was a significant effect of age (logistic regression: p = 0.0278) and a trend for transfer type (logistic regression: p = 0.051178) with younger participants and participants in the “matched” condition achieving more accurate discrimination. There were no significant effects for years of musical training, ability to speak a tonal language, or sex (logistic regression: all ps > 0.05). In octave six there was a significant effect of years of musical training and ability to speak a tonal language (logistic regression: both ps < 0.001) with more musically trained participants and speakers of tonal languages achieving more accurate discrimination. There were no significant effects for age, sex, or transfer type (logistic regression: all ps > 0.05). See Fig. 5 for a visualization of the response patterns in the respective groups.

### Additional analysis of generalization test data regarding vocal harmonic generalization

Additionally, we used Wilcoxon signed-rank tests to compare relative responses in the generalization test to notes and notes an octave plus perfect fifth above them – corresponding to the second overtone above the fundamental frequency in the human voice – in the -3+6-3 group generalization phase. We did this to test the hypothesis that as octave equivalence may be based on the harmonic series, participants may also generalize by the next most prominent interval in the harmonic series, the perfect fifth. There was no significant difference for responses to C#4 to G#6 (Wilcoxon: p = 0.2433), but for all other comparisons (Wilcoxon: C4 to G6: p = 0.031; D4 to A6: p < 0.001; D#4 to A#6: p < 0.005; E4 to B6: p < 0.001), with octave six notes being consistently responded to less.

We also used Wilcoxon signed-rank tests to test whether relative responses to notes that are an octave plus a perfect fifth apart from rewarded notes were different to relative responses to notes an octave plus a perfect fifth apart from unrewarded notes. We found no significant differences between responses to the rewarded range corresponding note A#6 and the non-rewarded range corresponding notes G6 (Wilcoxon: p = 0.6761), G#6 (Wilcoxon: p = 0.08631), and A6 (Wilcoxon: p = 0.9048).

**Fig. 6:**
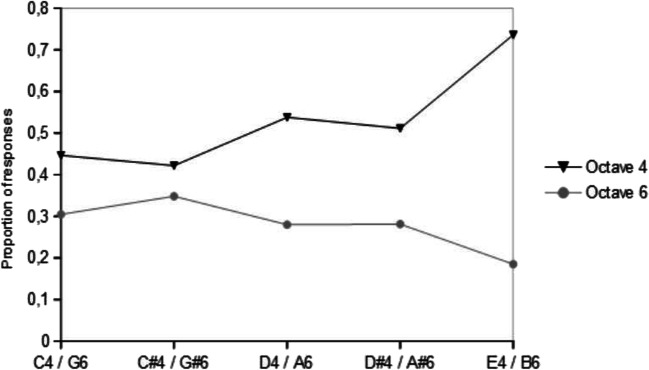
Mean proportion of responses to individual notes from octave four notes from the -3+6-3 group and octave six notes that are an octave plus a perfect fifth above them. The black line/triangle is octave four notes, grey line/circle is octave six notes

We found that there were significant differences between relative responses to the rewarded range corresponding note B6 and the non-rewarded range corresponding notes G6, G#6, and A6 (Wilcoxon: all ps < 0.01; this remains significant after Bonferroni correction at all ps < 0.05) with responses to B6 being consistently lower. See Fig. 6 for a visualization of these relationships.

## Discussion

Hoeschele et al. (2012a, b), as Kallman (1982) before them, suspected that pitch height perception may be dominant over that of octave equivalence in pitch identification tasks. Though octave equivalence is taken for granted in terms of musical tradition, a dominance of pitch height perception is the most likely reason why it has proven so difficult to show octave equivalence in experimental settings. Thus, we hypothesized here that if confounds of pitch height and octave equivalence – though perhaps they are not entirely avoidable – are not controlled for as rigorously as possible, pitch height perception will make the effects of octave equivalence negligible. This would explain why previous studies found conflicting results regarding human octave equivalence perception. The current experiment directly tested this hypothesis by using variations on the octave equivalence paradigm from Hoeschele et al. (2012a, b); a standardized non-musical and non-verbal operant paradigm that was shown to demonstrate an effect of octave equivalence in human adults. In particular, we implemented two-range tasks as well as the original three-range task, expecting participants to use pitch height to solve the two-range task and octave equivalence to solve the three-range task if our hypothesis regarding pitch height and octave equivalence was correct. Besides this, we were interested in whether octave equivalence could be demonstrated across more than one octave. The basis of octave equivalence may lie in the overtones of harmonic sounds such as the human voice, the first of which is the octave. If this is the case, generalization to higher overtones would be expected, but if this happens it is unclear whether it would be by an octave plus a perfect fifth (the second overtone in the human voice) or by two octaves (the third overtone). Finally, octave equivalence is often assumed to be cross-cultural and even universal. Yet, these claims are often more descriptive than empirical. As such, to gain a better perspective on these claims we analysed the participants’ questionnaire answers to illuminate potential effects of musicianship skills and spoken language on octave equivalence perception.

Results from the generalization phase are in line with our hypothesis that a two-range task fails to demonstrate octave equivalence because participants use pitch height and not octave equivalence to solve it. While the training pattern superficially appears to be replicated across octaves in the two-range groups, the two range types, S+ and S-, are not treated the same way across octaves. For example, in the -6+6 group, in every octave, participants respond more to the lower six notes compared to the upper six notes. However, responses to the S- ranges in octaves five and six are always higher than even the responses to the S+ ranges in octaves four and five, respectively. This suggests that participants in the -6+6 group were using the rule “respond more to high notes” rather than “respond to (e.g.) G notes”. In contrast, in the -3+6-3 group, participants responded comparably to S- and S+ ranges in octaves four and five and even partly in octave six. As such, participants appeared to only use pitch height in the two-range groups, and only use pitch chroma in the three-range group. Additionally, it is worth noting that the reward pattern in the three-range task does appear to be more difficult to learn because overall discrimination by participants in that task was lower. However, generalization testing results found the three-range group outperforming the other groups in octave four. This result may seem counter-intuitive, as the three-range group did not outperform the two-range group during discrimination. We suspect that it is probably due to the two-range groups using a ‘high’ versus ‘low’ pitch strategy – shifting their boundary for ‘high’ notes once two additional higher octaves were added. As such, for example, the -6+6 group treated all of octave four as S- once octave five was added. The three-range group, therefore, achieved relatively better accuracy in octave four exactly *because* they used pitch chroma.

Despite the three-range group appearing to use pitch chroma more readily than the two-range groups, results from the transfer test suggest that pitch chroma interferes in all reversed contingency transfer groups where notes that are rewarded differ in neighbouring octaves. All groups in reversed transfer appear to have discriminated less accurately. As such, even in the two-range groups where there was a trend towards the reversed condition’s rules being more difficult to learn, an effect of pitch chroma appears likely. This makes sense as the added octaves in combination with reward effectively turned the task from a two-range into a six-range task, which was most easily solvable by paying attention to pitch-chroma in the “non-reversed” condition.

This study added the important extension of moving from neighbouring octaves to more distant octaves to the paradigm from Hoeschele et al. (2012a, b). This addition was implemented to test the idea that the basis for octave equivalence may lie in the overtones of the human voice, the first of which corresponds to one octave above fundamental. While octave generalization was found between neighbouring octaves four and five in the three-range group, the picture was less clear for potential generalization by an octave plus a perfect fifth (which would correspond to the second overtone in the human voice) or by two octaves (which would correspond to the third overtone in the human voice). If participants had transposed by an octave plus a perfect fifth, we should expect responses to octave six notes A#6 and B6 to be higher than to G6, G#6, and A6 as the former would correspond to rewarded notes while the latter would correspond to unrewarded notes. However, out of those notes, B6 was least responded to. In addition to this, it does not appear that notes an octave plus a perfect fifth apart were perceived to be similar as responses were different for all such pairings except one. If participants had transposed by two octaves, responses in octave six should follow the pattern from octave four. If this is what happened, the generalization at most extended to the lower S- range of the -3+6-3 group. However, the -3+6-3 group octave six pattern could also be interpreted as responses simply declining with increasing pitch. Pitch perception appears to generally worsen with increasing pitch as even persons with absolute pitch fail by octave eight (Baharloo et al., 1998). Also, temporal pitch perception fails by around 2 kHz (Attneave & Olson, 1971). Octave six is relatively high pitched, being well beyond the usual pitch of human speaking and also of most singing. The connection to speaking pitch is particularly important with regards to the idea that octave equivalence is used in language learning (Peter et al., 2008, 2009, 2015) where vocal pitch usually does not differ by multiple octaves (see Wagner & Hoeschele, 2022, for a review). Thus, perhaps using a lower octave, closer to low human speaking pitch, as the training octave may have produced different results. Results found here suggest that if generalization happens across more than one octave using this paradigm it is by two octaves rather than an octave plus a perfect fifth.

Finally, another important finding of this study concerns the role of language with regards to octave equivalence perception. Experience with a tonal language, where pitch information may be more relevant – while appearing to have a positive effect when it comes to learning discriminations such as in the transfer test of the paradigm used here – had no effect on the generalization of the learned patterns across octaves. As such, octave equivalence from our data appears to be independent of language, a result that tentatively suggests that octave equivalence may occur similarly in different cultures. However, more research is needed, as all participants in this study were living in the Western hemisphere and had been exposed to Western music for a long time. We would like to emphasize at this point, that due to our participants being students at a predominantly English-speaking university, we could only consider the ability to speak a tonal language, but none were speaking a tonal language exclusively. Conceivably, participants that mostly or exclusively use a tonal language in their daily lives may differ in their performance on our task. Future studies could go beyond survey designs to test more directly for the role of language skills in octave equivalence perception and tone discrimination abilities. This could also allow for a new perspective on our data. Potentially, the effects of tonal language on perception of pitch chroma may be found to exist on a continuum and be more prevalent with exclusively tonal language speakers.

With regards to further research, this study offers additional insights. The results gathered in this paper add to an understanding of why humans perceive octave equivalence by way of understanding why the paradigm from Hoeschele et al. (2012a, b) succeeded in showing octave equivalence while others did not. While the paradigm from Hoeschele et al. (2012a, b) had already been replicated in the original study, the successful large sample size replication in this paper emphasizes the paradigm's potential as a reliable tool in documenting octave equivalence and gaining even further insights. A wide range of applications are possible, two of which we would like to point out.

One application regards studies in non-human animals. Avoiding the confounds of octave equivalence and pitch height appears to be just as paramount in studies with non-human animals as it does in humans (see, e.g., Hoeschele et al.’s (2012) discussion of Cynx (1993)). It bears repeating that the paradigm used herein can and has been implemented with non-human species, allowing for direct comparison with human results (Hoeschele et al., 2013; Wagner et al., 2019). Testing non-human animals for octave equivalence is of great interest because it makes it possible to control for cultural influences on the development of octave equivalence. It also allows us to examine what kind of ecological niches might result in the development of octave equivalence in a given species, giving us insight into its potential biological origins (see Hoeschele et al., 2015).

Another possible application of the paradigm is to use it with human participants where language barriers make other testing methods difficult. A recent study by Jacoby et al. (2019) found no effect of octave equivalence in the native Amazonian Tsimane’ tribe, a finding that has been important in the discussion about whether octave equivalence is truly a cross-cultural universal rather than being mostly or even entirely cultural. Jacoby et al. (2019) used an explicit task (a singing pitch-matching task) that they report not being able to verify with a non-verbal task due to difficulty replicating the results from Hoeschele et al. (2012a, b). Because, both through the original paper and the results reported here, the paradigm has been replicated several times within our group, Jacoby et al.'s report suggests that there may be other aspects to running this experiment that we are not aware of, which are critical for its success. Identifying what these aspects are may be critical to understanding when and under what conditions octave equivalence emerges. Non-verbal tasks like the current one are the ideal tool to study octave equivalence due to their applicability across cultures, human groups with language, or communication impairments, and across species. With enough groups working together on this problem, we may be able to directly address whether octave equivalence is truly a human universal.
